# Do we need a common liver fibrosis index or etiology-related indices?

**Published:** 2011-06-01

**Authors:** Mohamed Hessien

**Affiliations:** 1 Department of Chemistry, Section of Biochemistry, Faculty of Science, Tanta University, Tanta, Egypt

**Keywords:** Liver fibrosis, Chronic hepatitis C, Schistosoma mansoni

|Dear Editor,

Without downgrading the histological assessment of liver fibrosis, efforts are being made to develop accurate and reliable non-invasive indices as alternatives to liver biopsy. Previously proposed panels of liver fibrosis usually include 2 to 7 blood markers. The majority of publications were concerned with liver fibrosis among patients with chronic hepatitis C (CHC). However, few studies have assessed drug-mediated liver fibrogenesis, non-alcoholic fatty liver disease (NAFLD), Schistosoma mansoni-induced liver fibrosis, and hepatitis B virus-related liver fibrosis. In the work entitled "Noninvasive assessment of liver fibrosis with the aspartate transaminase to platelet ratio index (APRI): Usefulness in patients with chronic liver disease,"[1] authors have investigated 207 patients with chronic hepatitis B (CHB), 108 with CHC, and 140 with NAFLD. In this study, the APRI score [2] was calculated and matched with the histological stages of fibrosis using the METAVIR scale. Additionally, they employed the Kleiner system for grading the extent of fibrosis in NAFLD patients.

The authors came to the conclusion that APRI was significantly associated with fibrosis score in patients with CHC and NAFLD, but not in patients with CHB. However, some reports showed a significant correlation between fibrosis scores and APRI in Chinese [3] and Korean[4] patients with CHB. Although Yilmaz et al.[1] tried to explain the application of APRI in the assessment of hepatic fibrosis induced by different agents, they enhanced the contradictions and the misconceptions about using noninvasive markers implicated in the different indices. Previously reported indices have combined 1 (or more) variable of the following 4 categories: (i) extracellular matrix (ECM) proteins or enzymes (such as laminin, collagen, lysyl oxidase, prolyl hydroxylase, hyalauronic acid, or TIMP-1) which are known to play a role in scar formation, degradation, or activation of hepatic stellate cells (HSC) ; (ii) liver-specific markers (bilirubin, transaminases, or globulin); (iii) hematological variables (platelets or PT); and (iv) conjugated or derived lipids (such as: apolipoprotein and cholesterol). Recently, we combined reduced glutathione (GSH) as an oxidative stress marker [5] along with total bilirubin, gamma glutamyl transpeptidase (gGT), and total proteins. The validated index was able to monitor the progression and regression of fibrosis in fibrotic models generated through exposure to thiacetamide (TAA) and ethanol and infection with S. mansoni. Changes in the level of ECM proteins or the activity of ECM enzymes reported in some indices [6] usually comes next to the initial events of oxidative stress-mediated HSC activation. When Novitskiy et al.[7] treated HSC with ethanol, they noticed the formation of reactive oxygen species (ROS), which activated HSC and promoted fibrogenesis.

Consequently, studies that investigate populations with etiologically different liver fibrosis may require the use of indices that involve direct fibrogenesis and/or oxidative stress markers, in addition to liver specific markers. The limited number of variables in APRI may minimize its potential in monitoring the basic events in scar formation, which is the common scenario in etiologically different fibrosis. However, the contradictory outcomes of different studies in the last few years may raise the question: Do we need a common liver fibrosis index or etiology-related indices?
